# Non-coding RNAs in Natural Killer/T-Cell Lymphoma

**DOI:** 10.3389/fonc.2019.00515

**Published:** 2019-06-13

**Authors:** Mei Mei, Mingzhi Zhang

**Affiliations:** ^1^Department of Oncology, The First Affiliated Hospital of Zhengzhou University, Zhengzhou, China; ^2^The Academy of Medical Science, Zhengzhou University, Zhengzhou, China

**Keywords:** non-coding RNAs, microRNAs, EBV-encoded miRNAs, lncRNAs, natural killer/T-cell lymphoma (NKTCL)

## Abstract

Natural killer/T-cell lymphoma (NKTCL) is a rare and aggressive subtype of non-Hodgkin's lymphoma that is associated with a poor outcome. Non-coding RNAs (ncRNAs), which account for 98% of human RNAs, lack the function of encoding proteins but instead have the important function of regulating gene expression, including transcription, translation, RNA splicing, editing, and turnover. However, the roles and mechanisms of aberrantly expressed ncRNAs in NKTCL are not fully clear. Aberrant expressions of microRNA (miRNAs) affect the PI3K/AKT signaling pathways (miRNA-21, miRNA-155, miRNA-150, miRNA-142, miRNA-494), NF-κB (miRNA-146a, miRNA-155) and cell cycle signaling pathways to regulate cell function. Moreover, Epstein-Barr virus (EBV) encoded miRNAs and EBV oncoprotein LMP-1 regulated the expression of cellular genes that induce invasion, metastasis, cell cycle progression and cellular transformation. In addition, NKTCL-associated Long non-coding RNA (lncRNA) *ZFAS1* regulated certain pathways and lncRNA *MALAT1* acted as a predictive marker. This review article provides an overview of ncRNAs associated with NKTCL, summarizes the function of significantly differentially expressed hotspot non-coding RNAs that contribute to the pathogenesis, diagnoses, treatment and prognosis of NKTCL and discusses the relevance of these ncRNAs to clinical practice.

## Introduction

Non-Hodgkin's lymphoma (NHL) originates from B-lymphocytes, T-lymphocytes and natural killer (NK) lymphocytes and ranges from the indolent to the very aggressive (1). Each subtype could be further classified according to its origin, genetic signature or clinical features (2). Natural killer/T-cell lymphoma (NKTCL) is a rare and aggressive subtype of NHL that has a high incidence in East Asia and Latin America and that is associated with a poor outcome (3, 4). Extranodal NKTCL can be further classified into nasal NKTCL, which primarily affects the nasal cavity, nasopharynx and the upper aerodigestive tract, and non-nasal NKTCL, which involves the outside of the nasopharyngeal region, such as the skin, gastrointestinal tract and testis (5).

Deletion of chromosome 6q and down-regulation of tumor suppressor genes located on 6q21 regions, including *PRDM1* (6), *FOXO3* (7), *PTPRK, HACE1, ATG5*, and *AIM1* (8) were observed through oligo-array comparative genomic hybridization (CGH) and gene-expression profiling. High mutation frequencies of *FAS* (9), *TP53* and *DDX3X* (10) had a trend toward advanced stage and poor prognosis in NKTCL. Recurrent gene mutations in the JAK-STAT pathway were demonstrated, including high expression of phosphorylated-JAK3 and phosphorylated-STAT3 (11). Moreover, the expression of Epstein-Barr virus (EBV)-encoded RNA (EBER) was specific for diagnosis in clinical practice (12).

Non-coding RNAs (ncRNAs), which account for 98% of all human RNAs, lack a protein-coding function, but rather, they have the important function of regulating gene expression, including transcription, translation, RNA splicing, editing, and turnover (13). NcRNAs include microRNAs (miRNAs), small nuclear RNAs, PIWI-interacting RNAs, long non-coding RNAs (lncRNA), and circular RNAs. With the development of next-generation sequencing and bioinformatics approaches, ncRNAs show great biological importance in cancers. However, the roles and mechanisms of aberrantly expressed non-coding RNAs in NKTCL have not been fully clarified. This review article provides an overview of the recent advancements of ncRNAs associated with NKTCL and discusses their relevance to clinical practice.

## MiRNAs

MiRNAs are 18–24-nucleotide-long single-stranded ncRNAs that can regulate translation via binding to 3′-untranslated regions (3′-UTRs) of target mRNAs in order to affect cell function (14, 15).

## MiRNA-21

MiRNA-21 regulates various genes and signaling pathways involved in cancer pathogenesis, progression and metastasis (16). MiRNA-21 is overexpressed in various solid tumor types including breast, colon, lung, pancreas, prostate, and stomach (17) tumors and is also upregulated in hematological malignancies such as chronic lymphocytic leukemia (18), acute and chronic myeloid leukemia (19), diffuse large B-cell lymphoma (20), cutaneous T-cell lymphoma (21) and Hodgkin lymphoma (22). The expression of miRNA-21 was found to be higher in NK-cell lymphoma-derived cell lines and in samples of primary NKTCL compared with normal natural killer cells (23, 24). MiRNA-21 regulated apoptosis of NK-cell lymphoma cell lines via the PTEN/AKT signaling pathway, and the downregulation of miRNA-21 led to the upregulation of phosphatase and tensin homolog (*PTEN)*, programmed cell death 4 (*PDCD4*) and the downregulation of pAKT. *PTEN*, served as a multi-functional tumor suppressor, commonly lost in human cancer and negatively regulating AKT/PKB signaling pathway (25, 26). *PDCD4*, a tumor suppresser, inhibited neoplastic transformation and invasion (27–29). In addition, the proapoptotic protein Bim was found to be increased (23, 30). The role of miRNA-21 in the pathogenesis of NKTCL suggests that miRNA-21 can serve as a new biomarker or target in the treatment of NKTCL.

## MiRNA-155

MiRNA-155 is overexpressed in various hematological and solid malignancies (16). MiRNA-155 regulates inflammation, immune cells, and the differentiation and maturation of tumor cells (31). The expressions of miRNA-155 were higher in NK-cell lymphoma cell lines and primary NKTCL specimens than in normal NK cells (23, 24). Among various NK-cell lymphoma cell lines, the expression of miRNA-155 was reported to be highest in SNK-6 cells (32). MiRNA-155 regulated apoptosis via the PTEN/AKT and NF-κB signaling pathways in NK-cell lymphoma cell lines. MiRNA-155 directly down regulated Src homology-2 domain-containing inositol 5-phosphatase 1 (*SHIP1)*, which inhibited signaling in the phosphoinositide 3-kinase (PI3K)-AKT pathway and further inhibited p21 and p27 (23). Moreover, the expression of miRNA-155 was related to several inflammatory factors, such as interleukin 6 (IL-6), interleukin 13 (IL-13), and tumor necrosis factor (TNF), in NKTCL (33). In addition, whole DNA hypomethylation was observed to occur with locus-specific hypermethylation, especially on promoter-associated CpG islands, which resulted in the silencing of downstream genes and ncRNAs (34, 35). In NKTCL patients, the prevalence of miRNA-155-3p methylation has been discovered to occur distinctively with the overexpression of LT-β (35). MiRNA-155 is a potential molecular marker of NKTCL (33).

## MiRNA-142

MiRNA-142 has two different forms (miRNA-142-3p and miRNA-412-5p) that participate in the regulation of hematopoietic differentiation and immune response (36). MiRNA-142 upregulates various proteins such as the IL-6, interleukin 6 signal transducer (IL6ST), toll-like receptor 2 (TLR2), prostaglandin E receptor 2 (PGE2), and TNF (37). The miRNA-142-5p and miRNA-142-3p were under-expression in NKTCL compared with EBV-negative lymphomas (38). MiR-142-3p down regulated *RICTOR*, one of components of the mTOR complex, and further affected pAKT in YT cell line (39). In addition, the down-regulation of miR-142-3p led to the upregulation of IL1A in NKTCL (38). MiRNA-142-3p is a potential target of therapy (39).

## MiRNA-494

As a tumor suppressor miRNA, miRNA-494 played a role in various tumors (40, 41). MiRNA-494 induced *PTEN* downregulation in cervical cancer cells (42) and myeloid cells (43). In addition, TGF-β1 was a tumor-derived factor that was associated with the upregulation of miRNA-494 in MDSCs and MMPs, which led to tumor cell invasion and metastasis (43). In an NK-cell lymphoma cell line NK92, miRNA-494-3p was also found to down-regulate PTEN, which activated AKT in accordance with previous reports (39). Moreover, miRNA-494-3p worked in coordination with the EBV-encoded miRNA-BART20-5p, which inhibited the T-bet-PTEN pathway, with subsequent upregulation of AKT and suppression of *TP53* (39). Antagomir to miRNA-494-3p may serve as a potential target of therapy of NKTCL (39).

## MiRNA-150

MiRNA-150 as a key regulator of the differentiation and activation (44) of immune cells, such as B-, T-, and NK-lymphocytes (45), abnormally expressed in solid (46) and hematological malignancies (44). MiRNA-150 was found to be apparently lower in lymphoma cell lines and primary lymphoma specimens compared with normal NK cells, while no significant difference was found between resting and activated NK cells (24). Furthermore, miRNA-150 down regulated PIK3AP1 and AKT2, which were part of the PI3K-AKT pathway and upregulated Bim and p53. MiRNA-150 led to cancer cell anti-apoptosis and immortality, as pAKT^ser473/4^ acted on telomerase via phosphorylation of hTERT (24). In addition, miRNA-150 down regulated DKC1, which functioned in regulating pseudouridine in RNA and the telomerase RNA subunit hTR in NKTCL cells (24, 47). MiRNA-150 provides novel strategy upstream of AKT in the treatment of NKTCL (24).

## MiRNA-223

MiRNA-223 is strongly expressed in the bone marrow and bone marrow cells but is absent in B- and T-lymphocytes (48). In resting NK cells, miRNA-223 downregulated in the case of cytokine activation and controls GzmB translation in resting NK cells (49). Overexpression of miRNA-223 can decrease cancer cell proliferation (50, 51). For instance, miRNA-223 expression was reported to be lower in CD19^+^ lymphocytes in patients with mantle cell lymphoma compared with healthy donors (50). In NKTCL cells, overexpression of miRNA-223 is associated with cell differentiation (52). Positive regulatory domain containing I (*PRDM1*), a tumor suppressor gene in NK cell, was directly downregulated by miRNA-223 in NKTCL patient samples and NKTCL cell lines (53). All miRNA-223-positive samples from patients with NKTCL showed EBV infection, which implied that EBV infection may be responsible for miRNA-223 overexpression (53).

## MiRNA-16

Members of the miRNA-16 family function as tumor suppressors in a number of cancers via the regulation of the cell apoptosis pathway (54) and the cell cycle (55). In NK-cell lymphoma cell lines and primary tissue, miRNA-16 was found to be under expressed (56). MiRNA-16 and SAHA shared common therapeutic targets and induce senescence and apoptosis in NKTCL. However, in Kitadate's study, NK-cell lymphoma cell lines with non-functional p53 (KHYG1) did not show senescence caused by miRNA-16 or by SAHA. It has been confirmed that miRNA-16 or SAHA induces apoptosis, downregulates survivin and upregulates cleaved caspase-3 and *CDKN1A* (also known as p21) (56). This finding suggests that miRNA-16 has the potential to serve as a novel target in NKTCL treatment.

The miRNAs and their identified target genes are listed in Table 1.

**Table 1:** Summary of miRNAs and their target genes in NKTCL.

**miRNA**	**Targets**		**References**
	**Upregulation**	**Downregulation**	
**Overexpression of miRNAs in NKTCL**			
miRNA-21		*PTEN, PDCD4*	(23)
miRNA-155	*FOXO3*	*SHIP1*	(23, 33)
miRNA-223		*PRDM1*	(53)
miRNA-494		*PTEN*	(39)
**Under-expression of miRNAs in NKTCL**			
miRNA-150	*BIM, TP53*	*PIK3AP1*	(24)
miRNA-142		*RICTOR*	(39)
miRNA-30b		*PRDM1*	(57)
miRNA-15a	*MYB*		(58)
miRNA-148a		*CUL5*	(59)
miRNA-16		*CDKN1A*	(56)
MiRNA-146a		*TRAF6*	(60)

## EBV-Encoded MiRNAs

Since NKTCL is an EBV associated lymphoma, researchers illustrated that EBV infection promoted the progression from a lesion into NKTCL (33, 61, 62) via the regulation of 44 microRNAs (59). The most common outcome of EBV infection was viral latency, including type I, II and III (63). Latency I was reported to only express characterized EBNA-1, while latency II expressed EBNA-1 as well as LMP-1 and 2. Moreover, latency III with B cell infection expressed all EBNAs and LMPs (64). Furthermore, latency patterns were distinct in different malignant subtypes due to the expression of subsets of the latent genes (65, 66).

LMP-1, encoded by the *BNLF-1* gene, is the principal EBV oncoprotein and regulates the expression of cellular genes that induce invasion, metastasis, cell cycle progression and cellular transformation (63, 67, 68). LMP-1 mediates NF-κB and PI3K/AKT activation in EBV-positive NKTCL cell lines and inhibits cell apoptosis by promoting survivin expression (69–71). In addition, LMP1 also regulates cell function through regulation of the expression of other miRNAs; for instance, LMP1 inhibits the cell cycle via the downregulation of miRNA-15a, which inhibits MYB and cyclin D1 in NKTCL cells (58). In addition, EBV-miRNA-BART9 upregulates LMP1 (72).

Various studies have attempted to determine the clinical significance of miRNA-BARTs. In one study, the higher expression of miRNA-BART2-5p, miRNA-BART7-3p and miRNA-BART13-3p led to a poorer prognosis in patients with NKTCL (73). MiRNA-BART20-5p and miRNA-BART8 led to cell apoptosis via the inhibition of the IFN-γ-STAT1 pathway and the downregulation of miRNA-let7 in NKTCLs (65). Additionally, miRNA-BART20-5p inhibited *TP53* via T-bet (74, 75), a member of the T-box family that involved in tumor development (76). MiR-BART16 down-regulated the sphingosin-1-phosphate receptor 1 (*S1PR1*), which expressed in cells of lymphoid origin and named as CD363 antigen (59).

These studies have implications in the mechanisms of lymphomagenesis, and future experiments should be directed at the investigation of the role of EBV miRNAs and their regulation of cellular targets. The EBV-encoded miRNAs and their identified target genes are listed in Table 2 and associations are shown in **Figure 2**.

**Table 2:** Summary of EBV-encoded miRNAs and their targets in NKTCL.

**MiRNA**	**Targets**		**Targets function**	**References**
	**Upregulation**	**Downregulation**		
miRNA-BART-20		*T-bet*	Terminal maturation of NK cell	(65)
miRNA-BART-8		*STAT1*	Induce apoptosis	(65)
miRNA-BART-16		*S1PR1*	Induce cell-cell adhesion	(59)
miRNA-BART-9	*BNLF-1* (protein LMP-1)		Induce cell proliferation in NKTCL	(72)

## Other miRNAs

In NK-cell lymphoma cell lines, various miRNAs function in the regulation of tumor development (77), including miRNA-101, miRNA-26a, miRNA-26b, miRNA-28-5, and miRNA-363 (57).

MiRNA-20, miRNA-26a, miRNA-92, miRNA-103, and miRNA-181 were shown to be overexpressed in patients of NKTCL (23). Moreover, miRNA-424 (38) and miRNA-16 (56) were shown to be under-expressed in NK-cell lymphoma cell lines and tumor tissue. The expression of miRNA-221 in the serum of NKTCL patients might be a prognostic factor since high expression leads to a poorer overall survival (OS) (78).

Furthermore, miRNAs regulate gene expression. For example, the expression of *PRDM1* was directly downregulated by miRNA-30b in NKTCL (57). In addition, CUL5 is a target of deregulated miRNA-148a in NKTCL (59).

MiRNA-15a was reported to inhibit the cell cycle by blocking G1/S progression in NK-cell lymphoma cell lines (58). Specifically, miRNA-15a upregulated MYB and cyclin D1 which were essential for the proliferation of NK-cell lymphoma cells (58).

As a tumor suppressor, miRNA-34a (79) was found to be hypermethylated in both myeloma and lymphoma cell lines (80). Furthermore, in lymphoma primary patient samples, methylation of miRNA-34a was found to be more frequently in NKTCL than in B- or T-cell lymphoma (80).

MiRNA-146a also exhibited hypermethylation in NKTCL, and down-regulated its target gene *TRAF6* and NF-κB signaling pathway (60). In clinical study, low miRNA-146a expression was an independent poor prognostic factor.

**Figure 1:**
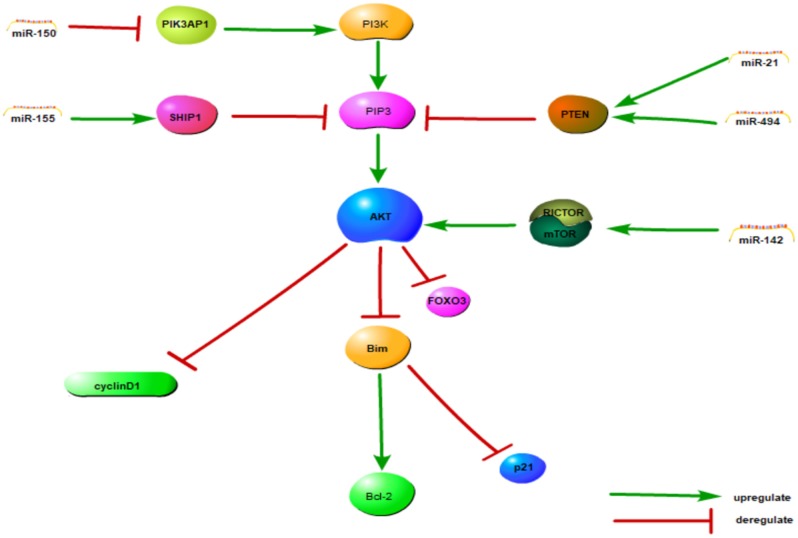
The PI3K/AKT and cell cycle signaling pathway model of NKTCL. MiRNAs can influence almost every cellular behavior from transcription to translation by diverse mechanisms including the cell cycle and the PI3K/AKT pathways, among others. All the miRNAs in this figure are upregulated in NKTCL sample, except miRNA-150. This figure adapted from Chen et al. (39) and the appropriate copyright permissions has been obtained from the copyright holder of this work.

In summary, we found that dysregulation of miRNAs might be a key feature of the pathogenesis of NKTCL. Aberrant expression of miRNAs might affect the AKT, NF-κB and cell cycle signaling pathways to regulate cell function. The signaling pathway model has been integrated in Figure 1. Hypermethylation is another way by which cell function is regulated. These findings provide new thought about the pathogenesis of NKTCL.

## LncRNAs

LncRNAs are a group of RNAs >200 nucleotides in length that regulate gene expression by transcriptional and posttranscriptional destabilization (81, 82). LncRNAs have great potential value in the pathogenesis, diagnosis, treatment and prognosis of malignant tumors (83, 84).

Baytak et al. (85) conducted whole transcriptome sequencing (WTS) analysis on NKTCL cases, normal NK-cells and NK-cell lymphoma cell lines. They revealed 166 lncRNAs with more than 1.5-fold overexpression, such as RAB30-AS1, ARAP-AS1 and PRMT5-AS1 which may have biological function on cell growth.

**Figure 2:**
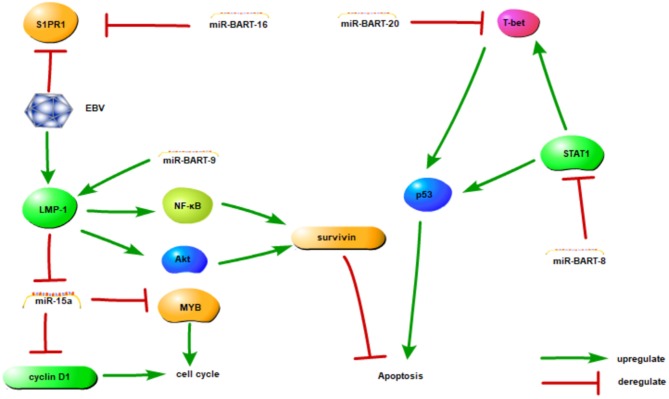
The association between EBV-miRNAs and targets in NKTCL. This figure adapted from Huang and Lin (65) and the appropriate copyright permission has been obtained.

LncRNA *ZNFX1* antisense RNA 1 (*ZFAS1*) transcribed from the antisense gene *ZNFX1* (86), was overexpressed in mammary gland while under-expressed in breast tumors (87). However, *ZFAS1* functioned as oncogene in various tumors, such as glioma (88), colorectal cancer (89), Gastric cancer (90), hepatocellular carcinoma (91) and ovarian cancer (92) for the high expression in tumor tissues. Moreover, *ZFAS1* also showed high expression in AML cell lines (93). LncRNA *ZFAS1* was observed to be upregulated in NKTCL and further demonstrated 483 relevant genes (70 genes with strong positive correlation, while 413 genes with strong negative correlation). Overall, ZFAS1-correlated genes associated with the upregulation of certain pathways, including non-sense-mediated mRNA decay, NF-κB signaling, β-catenin independent WNT signaling and p53-dependent apoptosis and the cell cycle pathways (Figure 2) (85). In addition, lncRNA *ZFAS1* regulates p53 via invasion and metastasis related genes MDM2, and p53 can further regulate the NF-κB, WNT, and NOTCH1 pathways (85).

LncRNA metastasis-associated lung adenocarcinoma transcript 1 (*MALAT1*), also known as nuclear-enriched transcript 2 (*NEAT2*), is discovered as a predictive marker for metastasis and survival in early-stage, non-small cell lung cancer (94). The high expression of *MALAT1* have been found in various cancer types (95, 96) and predicted metastasis or poor prognosis (97, 98). LncRNA *MALAT1* was highly expressed in NKTCL, but in the absence of expression, inferior OS is observed. *MALAT1* was important in sustaining PRC2-induced H3K27me3, which led to the subsequent activation of *BMI1* which predicted the clinically aggressive behaviors in NKTCL (99, 100).

The functions and regulations of lncRNAs were not isolated. Positive regulatory domain containing I (*PRDM1*), which acted as a tumor suppressor gene, was silenced in NKTCL as previously mentioned (7). *PRDM1*α regulated 212 lncRNAs (169 upregulation and 103 downregulation). Among them, MIRNA-155HG and *TERC* may be an indirect target of *PRDM1* in NK-cell lymphoma cells. However, biological functions of most lncRNAs regulated by *PRDM1* in NKTCL remained unknown, and further studies should involve functional characterization (85).

## Conclusion

With the development of next-generation sequencing and bioinformatics, non-coding RNAs have shown promising value in tumor research in recent years. However, the focus on B-cell and T-cell lymphoma encompasses much more than NKTCL. This article summarizes the function of significantly differentially expressed hotspot non-coding RNAs that contribute to the pathogenesis, diagnoses, treatment and prognosis of NKTCL. The detailed mechanisms and the function of the non-coding RNAs mentioned above still remain to be clarified. Further connections among these non-coding RNAs can be supplemented. Moreover, research on novel non-coding RNAs such as piwi RNAs, circular RNAs and tiRNAs that are associated with NKTCL is needed. Clinical research of thoroughly studied non-coding RNAs could be performed and lead to the early diagnoses of NKTCL and could be useful in drug resistance or targeted therapy.

## Author Contributions

MZ and MM designed the study. MM collected data and wrote the manuscript. All authors read and approved the final manuscript.

### Conflict of Interest Statement

The authors declare that the research was conducted in the absence of any commercial or financial relationships that could be construed as a potential conflict of interest.

## Abbreviations

NHL: Non-Hodgkin's lymphoma
EBER: EBV-encoded RNA
NKTCL: Natural killer/T-cell lymphoma
EBV: Epstein-Barr virus
ncRNAs: non-coding RNAs
CGH: comparative genomic hybridization
miRNAs: microRNAs
3′-UTRs: 3′ - untranslated regions
IL-6: immune cytokines interleukin 6
IL6ST: interleukin 6 signal transducer
TLR2: toll-like receptor 2
PGE2: prostaglandin E receptor 2
TNF: tumor necrosis factor
OS: overall survival
WTS: whole transcriptome sequencing
*PTEN*: phosphatase and tensin homolog
*PDCD4*: programmed cell death 4
*SHIP1*: Src homology-2 domain-containing inositol 5-phosphatase 1
*PI3K*: phosphoinositide 3-kinase
*PRDM1*: Positive regulatory domain containing I
*S1PR1*: sphingosin-1-phosphate receptor 1
*ZFAS1*: ZNFX1 antisense RNA 1
*MALAT1*: metastasis-associated lung adenocarcinoma transcript 1
*NEAT2*: nuclear-enriched transcript 2.
